# Characteristics of Carbapenem-Resistant *Enterobacteriaceae* in Ready-to-Eat Vegetables in China

**DOI:** 10.3389/fmicb.2018.01147

**Published:** 2018-06-01

**Authors:** Bao-Tao Liu, Xiao-Yan Zhang, Shu-Wei Wan, Jun-Jie Hao, Rui-De Jiang, Feng-Jing Song

**Affiliations:** ^1^College of Veterinary Medicine, Qingdao Agricultural University, Qingdao, China; ^2^Institute of Plant Protection, Qingdao Academy of Agricultural Sciences, Qingdao, China

**Keywords:** characteristics, *Enterobacteriaceae*, carbapenemase, plasmids, vegetables

## Abstract

Vegetables harboring bacteria resistant to antibiotics are a growing food safety issue. However, data concerning carbapenem-resistant *Enterobacteriaceae* (CRE) in ready-to-eat fresh vegetables is still rare. In this study, 411 vegetable samples from 36 supermarkets or farmer's markets in 18 cities in China, were analyzed for CRE. Carbapenemase-encoding genes and other resistance genes were analyzed among the CRE isolates. Plasmids carrying carbapenemase genes were studied by conjugation, replicon typing, S1-PFGE southern blot, restriction fragment length polymorphism (RFLP), and sequencing. CRE isolates were also analyzed by pulsed-field gel electrophoresis (PFGE). Ten vegetable samples yielded one or more CRE isolates. The highest detection rate of CRE (14.3%, 4/28) was found in curly endive. Twelve CRE isolates were obtained and all showed multidrug resistance: *Escherichia coli*, 5; *Citrobacter freundii*, 5; and *Klebsiella pneumoniae*, 2. All *E. coli* and *C. freundii* carried *bla*_NDM_, while *K. pneumoniae* harbored *bla*_KPC−2_. Notably, *E. coli* with *bla*_NDM_ and ST23 hypervirulent *Klebsiella pneumoniae* (hvKP) carrying *bla*_KPC−2_ were found in the same cucumber sample and clonal spread of *E. coli, C. freundii*, and *K. pneumoniae* isolates were all observed between vegetable types and/or cities. IncX3 plasmids carrying *bla*_NDM_ from *E. coli* and *C. freundii* showed identical or highly similar RFLP patterns, and the sequenced IncX3 plasmid from cucumber was also identical or highly similar (99%) to the IncX3 plasmids from clinical patients reported in other countries, while *bla*_KPC−2_ in *K. pneumoniae* was mediated by similar F35:A-:B1 plasmids. Our results suggest that both clonal expansion and horizontal transmission of IncX3- or F35:A-:B1-type plasmids may mediate the spread of CRE in ready-to-eat vegetables in China. The presence of CRE in ready-to-eat vegetables is alarming and constitutes a food safety issue. To our knowledge, this is the first report of either the *C. freundii* carrying *bla*_NDM_, or *K. pneumoniae* harboring *bla*_KPC−2_ in vegetables. This is also the first report of ST23 carbapenem-resistant hvKP strain in vegetables.

## Introduction

The food chain has attracted public attention not only because the contamination of pathogens but also it can serve as a reservoir for resistance genes. Several studies have investigated the prevalence of antibiotic-resistant bacteria in the food chain around the world, especially in retail meat (Leverstein-van Hall et al., 2011; Belmar Campos et al., 2014; Petternel et al., 2014; Wu et al., 2015; Xie et al., 2016). Fresh vegetables can be also a threat to public health because outbreaks of foodborne diseases linking with contaminated vegetables have been increasing in recent years (Jung et al., 2014). Besides zoonotic pathogens, the commensal and environmental bacteria in contaminated vegetables can even serve as a reservoir for antibiotic resistance genes, prompting fresh vegetables to be a growing food safety issue (Zurfluh et al., 2015a). The contamination of bacteria can occur not only through animal manure fertilization, soil, and irrigation water, but also by washing, handling, and processing vegetables during post-harvest period (Berger et al., 2010; Seo and Matthews, 2014). In fresh vegetables, commensal *Enterobacteriaceae* such as *E. coli* are the biggest issue because of the antimicrobial-resistance among them, and some even caused outbreaks of foodborne diseases (Friesema et al., 2008; Edelstein et al., 2014), including the contaminated Shiga toxin-producing *Escherichia coli* (STEC) producing extended-spectrum β-lactamase (ESBL) in sprouts causing the outbreak in Germany in 2011 (Buchholz et al., 2011). Thereafter, ESBL-producing *Enterobacteriaceae* in vegetables were reported in several countries (Veldman et al., 2014; Zurfluh et al., 2015a; Luo et al., 2017; Mesbah Zekar et al., 2017; Randall et al., 2017).

The emergence of carbapenem-resistant *Enterobacteriaceae* (CRE) is of great concern to public health, and CRE isolates have been found in samples of different origins around the world in recent years, including humans (Singh-Moodley and Perovic, 2016; Lodise et al., 2017; Zhang et al., 2017), hospital wastewater (Lamba et al., 2017), animals (Liu et al., 2017), seafood products (Morrison and Rubin, 2015), and retail meat (Wang et al., 2017). In fresh vegetables, one *Klebsiella variicola* producing OXA-181 and three *Klebsiella pneumoniae* producing OXA-48 were found in Switzerland (Zurfluh et al., 2015b) and Algeria (Touati et al., 2017), respectively. Notably, only one *E. coli* co-producing NDM-1 and KPC-2 carbapenemases was recently reported in lettuce in Guangzhou, China (Wang et al., 2018). However, studies focusing on carbapenemase-producers in contaminated fresh vegetables were still few. Considering the high occurrence of CRE in different origins in China including humans (Zhang et al., 2017), animals (Liu et al., 2017), and retail meat (Wang et al., 2017), there is an urgent need to investigate the prevalence of CRE in vegetables, especially ready-to-eat fresh vegetables. There is also a need to investigate the CRE besides *K. variicola, K. pneumonia*, and *E. coli* in vegetables in China.

In this study, we conducted a surveillance of the prevalence of CRE in fresh vegetables in China and investigated the molecular epidemiological features of these strains. Findings of this work shall provide essential insight into development of effective strategies for control of CRE in food and reducing untreatable infections in clinical settings.

## Methods

### Sampling

Between May and Nov 2017, 17 different types of fresh vegetables were purchased from 36 supermarkets or farmer's markets in 18 cities or districts of 7 provinces (Beijing, Tianjin, Shanghai, Shandong, Henan, Jiangsu, and Heilongjiang) in China (Table S1). In total, 411 samples from fresh vegetables were collected for analysis. The 411 vegetable samples included cucumber (*Cucumis sativus* L., *n* = 74), tomato (*Lycopersicon esculentum* Mill., *n* = 67), romaine lettuce (*Lactuca sativa* L., *n* = 35), green pepper (*n* = 34), leaf rape (*Brassica napus* L., *n* = 33), curly endive (*Cichorium endivia L., n* = 28), chili pepper (*Capsicum annuum* L., *n* = 22), spinach (*Spinacia oleracea* L., *n* = 22), mungbean sprouts (*Vigna radiata* L., *n* = 21), coriander (*Coriandum sativum* L., *n* = 19), leaf lettuce (*Lactuca sativa var longifoliaf*. Lam, *n* = 1 6), pakchoi (*Brassica chinensis* L., *n* = 10), carrot (*Daucus carota* L., *n* = 9), soybean sprouts (*Glycine max* L. Merr., *n* = 8), garland chrysanthemum (*Chrysanthemum coronarium* L., *n* = 5), eggplant (*Solanum melongena* L., *n* = 4), and green shallots (*Allium ascalonicum* L., *n* = 4). All samples were collected in sterile containers, stored under refrigeration, and processed within 24 h.

### Identification of CRE isolates and carbapenemase-encoding genes

Of each unwashed sample, 10 g was placed aseptically in a sterile flask containing 90 ml of trypticase soy broth (Becton Dickinson, Breda, the Netherlands) supplemented with meropenem (1.0 mg/L) and vancomycin (8 mg/L), and then was shaken vigorously (incubation overnight at 37°C). Vancomycin was added to the broth to ensure inhibition of the growth of Gram-positive bacteria. Thereafter, 50 mL of the sample suspension with survived bacteria was centrifuged at 6,000 × g for 10 min at 4°C. The obtained pellets were weighted (200 mg per sample) and extraction of the total DNA from each pellet was performed using a MoBio Powersoil DNA isolation kit (MoBio) following the manufacturer's instructions. The remaining broth with survived bacteria was diluted in series of 1:10 and an aliquot (100 μL) of appropriate dilution was spread onto CHROMagar KPC plates (CHROMagar, Paris, France) which were prepared according to the manufacturer's instructions, followed by incubation for 18 h at 37°C. As different colonies might exist in the same sample, 3 colonies with the same *Enterobacteriaceae* appearance on one CHROMagar KPC plate were selected for PFGE subtyping. The CRE isolates obtained were identified by the typical appearances on plate and confirmed by 16S rRNA sequence and *rpoB* sequence analysis (Mollet et al., 1997). The total DNA of sample-processed broth and DNA of confirmed CRE isolates were screened for carbapenemase-encoding genes *bla*_IMP_, *bla*_VIM_, *bla*_NDM_, *bla*_SPM_, *bla*_AIM_, *bla*_DIM_, *bla*_GIM_, *bla*_SIM_, *bla*_KPC_, *bla*_BIC_, and *bla*_OXA−48_ using primers previously described (Poirel et al., 2011). The presence of these genes was confirmed by sequencing obtained amplicons. To identify the subtypes of *bla*_NDM_, the complete coding sequence was amplified and sequenced using reported primers (Zong and Zhang, 2013).

### Antimicrobial susceptibility testing

The minimal inhibitory concentrations (MICs) of 20 antibiotics, namely cefotaxime, ceftiofur, ceftazidime, ertapenem, imipenem, meropenem, ampicillin, enrofloxacin, ciprofloxacin, levofloxacin, nalidixic acid, amikacin, gentamicin, kanamycin, streptomycin, doxycycline, tetracycline, tigecycline, fosfomycin, and florfenicol were assayed by the agar dilution method, according to the guidelines of the Clinical and Laboratory Standards Institute (CLSI) (CLSI, 2015b). The breakpoints for each antimicrobial drug except tigecycline were recommended by the CLSI (CLSI, 2015a,b). The MIC method for colistin and resistant breakpoints for colistin and tigecycline were recommended by the 2017 EUCAST (available at http://www.eucast.org/clinical_breakpoints/). *E*. *coli* ATCC 25922 was used as the control strain. Multidrug resistance was defined as non-susceptibility to at least one agent in three or more antimicrobial categories (Magiorakos et al., 2012).

### Detection of other resistance genes

The CRE isolates were screened for the presence of PMQR (*qnrA, qnrB, qnrS, qnrC, qnrD, qepA, oqxA*, and *oqxB*), *bla*CTX-M and *fosA3* genes by PCR (Briñas et al., 2003; Weill et al., 2004; Liu et al., 2007, 2011; Yue et al., 2008; Cavaco et al., 2009; Wang et al., 2009). 16S rRNA methyltransferase genes (*armA, rmtA, rmtB, rmtC, rmtD, npmA*, and *rmtE*) among the CRE isolates were detected by PCR as previously described (Doi and Arakawa, 2007; Wachino et al., 2007; Davis et al., 2010). The presence of transferable colistin resistance genes (*mcr-1* to *mcr-7*) was also determined using primers previously described (Liu et al., 2016; AbuOun et al., 2017; Rebelo et al., 2018; Yang et al., 2018).

### MLST and PFGE typing

Multilocus sequence typing (MLST) of the *E. coli* isolates was performed as previously described (Wirth et al., 2006). Sequences were imported into the *E. coli* MLST database website (http://mlst.ucc.ie/mlst/dbs/Ecoli) to determine MLST types. MLST of the *K. pneumoniae* isolates was determined according to the previously described method (Diancourt et al., 2005). Sequence types (STs) were determined according to the MLST database website (http://bigsdb.pasteur.fr/klebsiella/submission_mlst.html). Clonal relationships of all CRE isolates were also investigated by PFGE of *Xba*I-digested genomic DNAs as previously described (Gautom, 1997). The *Xba*I-digested DNA of *Salmonella* Braenderup strain H9812 was used as a molecular weight marker. The PFGE patterns were analyzed with BioNumerics software version 2.5 (Applied Maths) to describe the relationships of the test strains.

### Conjugation experiments

Conjugation experiments were performed using the broth-mating method as previously described (Chen et al., 2007). *E. coli* C600 (streptomycin resistant) was used as the recipient. Transconjugants were selected on MacConkey agar plates containing streptomycin (2000 mg/L) and meropenem (1.0 mg/L). The transconjugants were confirmed by PCRs mentioned above and Enterobacterial repetitive intergenic consensus PCR (ERIC-PCR) previously described (Versalovic et al., 1991). The transconjugants should show the same ERIC-PCR patterns with the recipient.

### Plasmid analysis of transconjugants

Among the transconjugants, Incompatibility (Inc) groups were assigned by the PCR-based replicon typing method (Carattoli et al., 2005). The IncX and IncI2 replicons were detected according to previous methods (Johnson et al., 2012; Chen et al., 2013). To analyze the location of the carbapenemase-encoding genes of transconjugants, S1 nuclease-PFGE was performed twice as previously described (Barton et al., 1995). Subsequently, Southern blot hybridization was performed repeatedly with DNA probes specific for *bla*_NDM_ or *bla*_KPC−2_ which were prepared using the purified products of obtained PCR amplicons and were non-radioactively labeled with a DIG High Prime DNA labeling and detection kit (Roche Diagnostics, Mannheim, Germany). *E. coli* isolate harboring plasmids without *bla*_NDM_ was used to prove the specific for *bla*_NDM_. Plasmid DNA extraction was performed using a QIAGEN Plasmid Midi kit (QIAGEN, Germany). Plasmids of transconjugants were digested with the endonuclease *EcoR*I (TaKaRa Biotechnology, Dalian, China) to analyze the RFLP profiles.

To investigate the genetic characteristic of prevalent plasmids harboring *bla*_NDM_, representative plasmid was selected for sequencing. Briefly, the total genomic DNA from transconjugants was extracted using the Wizard Genomic DNA Purification kit (Promega), and then sequenced using both the Illumina Hiseq platform and the Pacbio RS platform. After assembling the sequence reads and filtering the data of C600 chromosomal DNA, contigs of the plasmid were obtained. The RAST annotation pipeline was chosen to perform rapid annotation of the plasmid (Overbeek et al., 2014). Comparison of the plasmid against the highly homologous plasmids in the NCBI database was performed by BRIG (Alikhan et al., 2011).

## Results

### Prevalence of carbapenemase-encoding genes and CRE isolates in ready-to-eat vegetables

In total, ten (2.4%) of the 411 vegetable samples were found to carry carbapenemase-encoding genes when screening the total DNA of broth, including 4 samples with *bla*_NDM−5_, 5 with *bla*_NDM−1_, and 2 with *bla*_KPC−2_. Notably, one cucumber sample was found to carry *bla*_NDM−5_ and *bla*_KPC−2_, simultaneously. Twelve CRE isolates were retrieved from the 10 samples using the CHROMagar KPC plates and all produced carbapenemases (Table 1). VS1 and VS2 with identical PFGE pattern were from the same romaine lettuce sample, however, *oqxAB* was found in VS2. Analysis of 16S rRNA sequences and *rpoB* sequences of these CRE isolates showed that the number of *E. coli, Citrobacter freundii*, and *Klebsiella pneumoniae* was 5 (35.7%), 5 (35.7%), and 2 (14.3%), respectively (Table 1). All 12 CRE isolates carried one carbapenemase gene and no difference was found in carbapenemase genes content between the direct analysis of the total DNA of broth and the analysis of CRE isolates grown on the selective plates. *bla*_NDM_ was the dominant type and found in 10 isolates (5 *E. coli* isolates carrying *bla*_NDM−5_ and 5 *C. freundii* isolates with *bla*_NDM−1_), whereas *bla*_KPC−2_ was only found in the 2 *K. pneumoniae* isolates (Table 1). The “string test” was also performed on the 2 *K. pneumoniae* isolates and *K. pneumoniae* strains with a positive string test (a viscous string longer than 5 mm could be generated by touching and pulling a single colony upwards with a standard inoculation loop) were designated hvKP (Liu et al., 2014). K1, K2, K5, K20, K54, and K57 serotypes were also analyzed as previously described (Fang et al., 2007). Both two KPC-2-producing *K. pneumoniae* isolates in this study were hvKP and also positive for K1 serotype. Of note, two CRE isolates with different morphologies (*E. coli* VH1 with *bla*_NDM−5_ and hvKP VH1-2 harboring *bla*_KPC−2_) were obtained from the cucumber sample carrying both *bla*_NDM−5_ and *bla*_KPC−2_. No other carbapenemase gene was found in this study.

**Table 1:** Characteristics of Carbapenem-resistant *Enterobacteriaceae* isolates from vegetables in China.

**Organism and isolate (ST types)^#^**	**Source**	**Market/City**	**Resistance phenotypes**	**Resistance genes**
***E. coli***				
VS1 (ST4762)	Romaine lettuce	Farmer's market A/LiCang	AMP, MEM, CTX, CTF, CAZ, ETP, IPM, NAL, ENR^*^, KAN, GEN, TET, DOX, FOS, FFC	*bla*_NDM−5_, *fosA3, bla*_CTX−M−1G_, *floR*
VS2 (ST4762)	Romaine lettuce	Farmer's markets A/LiCang	AMP, MEM, CTX, CTF, CAZ, ETP, IPM, NAL, ENR^*^, KAN, GEN, TET, DOX, FOS, FFC	*bla*_NDM−5_, *fosA3, bla*_CTX−M−1G_, *floR, oqxAB*
VH1 (UT)	Cucumber	Farmer's market A/ LiCang	AMP, MEM, CTX, CTF, CAZ, ETP, IPM, NAL, CIP, ENR, KAN, GEN, TET, DOX, FOS, FFC	*bla*_NDM−5_, *fosA3, floR, qnrB*
VH3-1 (ST4762)	Cucumber	Supermarket F/JiMo	AMP, MEM, CTX, CTF, CAZ, ETP, IPM, NAL, ENR, KAN, TET, DOX, FOS, FFC	*bla*_NDM−5_, *fosA3, bla*_CTX−M−1G_, *floR*,
VK70 (ST167)	Curly endive	Supermarket E/ BinZhou	AMP, MEM, CTX, CTF, CAZ, ETP, IPM, NAL, CIP, ENR, LEV, STR, KAN, GEN, AMK, TET, DOX, FOS, FFC	*bla*_NDM−5_, *fosA3, bla*_CTX−M−1G_, *floR, rmtB*
***C. freundii***				
VK5	Curly endive	Supermarket B/ LaiYang	AMP, MEM, CTX, CTF, CAZ, ETP, IPM, KAN, FOS, FFC	*bla*_NDM−1_, *fosA3, floR, qnrB*
VS7	Romaine lettuce	Supermarket B/ LaiYang	AMP, MEM, CTX, CTF, CAZ, ETP, IPM, NAL, ENR^*^, KAN, FOS, FFC	*bla*_NDM−1_, *fosA3, floR, qnrB*
VX9	Tomato	Supermarket C/ LaiYang	AMP, MEM, CTX, CTF, CAZ, ETP, IPM, NAL, CIP, ENR, LEV^*^, STR, KAN, GEN, TET, DOX, FOS, FFC	*bla*_NDM−1_, *fosA3, bla*_CTX−M−1G_, *floR, qnrB*
VK44	Curly endive	Supermarket D/ YanTai	AMP, MEM, CTX, CTF, CAZ, ETP, IPM, NAL, ENR^*^, STR, KAN, GEN, AMK, TET, DOX, FOS, FFC	*bla*_NDM−1_, *fosA3, floR, oqxAB, qnrB, rmtB*
VK49	Curly endive	Supermarket D/ YanTai	AMP, MEM, CTX, CTF, CAZ, ETP, IPM, NAL, ENR, STR, KAN, GEN, TET, DOX, FOS, FFC	*bla*_NDM−1_, *fosA3, floR, qnrB*
***K. pneumoniae***				
VH1-2 (ST23)	Cucumber	Farmer's market A/ LiCang	AMP, MEM, CTX, CTF, CAZ, ETP, IPM, NAL, ENR^*^, KAN, FOS	*bla*_KPC−2_, *qnrB, oqxAB*
VH11 (ST23)	Cucumber	Supermarket C/ LaiYang	AMP, MEM, CTX, CTF, CAZ, ETP, IPM, NAL, ENR^*^, KAN, FOS	*bla*_KPC−2_, *bla*_CTX−M−1G_, *qnrB, oqxAB*

# *Isolates VS1 and VS2 were isolated from the same sample. Isolates VH1, and VH1-2 were from the same sample. UT, Untypable*.

* *Intermediate resistance*.

*AMP, ampicillin; MEM, meropenem; CTX, cefotaxime; CTF, ceftiofur; CAZ, Ceftazidime; ETP, ertapenem; IPM, imipenem; NAL, nalidixic acid; CIP, ciprofloxacin; ENR, enrofloxacin; LEV, levofloxacin; STR, streptomycin; KAN, kanamycin; GEN, gentamicin; AMK, amikacin; TET, tetracycline; DOX, doxycycline; FOS, fosfomycin; FFC, florfenicol*.

The 10 samples with CRE belonged to 4 types of fresh vegetables and were all from Shandong province (Table 1, Table S1). The highest detection rate of CRE was found in curly endive (4/28, 14.3%) and the 4 samples were from 3 supermarkets in 3 cities (Binzhou, Laiyang, and Yantai) (Table 1, Table S1). City Laiyang was near to Yantai, while Binzhou was far away from both the two cities. Of the 35 romaine lettuce samples, 2 (5.7%) harbored CRE isolates, and they were from a farmer's market in City LiCang and a supermarket in City LaiYang (Table 1, Table S1). Among the 74 cucumber samples, 3 (4.1%) harbored CRE isolates and they were recovered from 2 supermarkets and 1 farmer's market in 3 cities. One of the 67 tomato samples (1.5%) carried CRE isolate. Of the 18 cities included in this study, the highest detection rate of CRE was found in Yantai (18.2%, 2/11), followed by Laiyang (12.9%, 4/31), Licang (10%, 2/20), Jimo (7.1%, 1/14), and Binzhou (6.7%, 1/15). None of the fresh samples in other 6 provinces carried CRE isolates in this study.

### PFGE and MLST types

**Figure 1:**
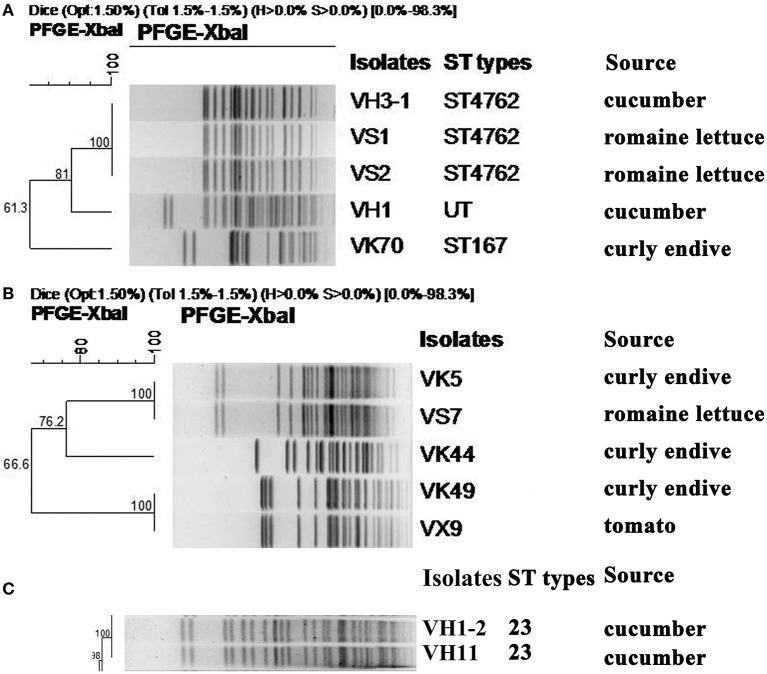
*Xba*I-PFGE patterns and sources of CRE isolates recovered from ready-to-eat fresh vegetables. **(A)** MLST and PFGE-based dendrogram of 5 *E. coli* isolates carrying *bla*_NDM_. UT, Untypable. **(B)** PFGE-based dendrogram of 5 *C. freundii* isolates carrying *bla*_NDM_. **(C)** MLST and PFGE-based dendrogram of 2 *K. pneumoniae* isolates carrying *bla*_KPC−2_.

Among the 5 carbapenem-resistant *E. coli* isolates, 3 different PFGE patterns were obtained (Figure 1). Notably, isolates VH3-1 and VS1 from cucumber and romaine lettuce samples in different cities, respectively, shared identical PFGE pattern and ST type (Figure 1, Table 1). Isolate VK70 from curly endive belonged to ST167.

In the 5 carbapenem-resistant *C. freundii* isolates, 3 different PFGE patterns were obtained (Figure 1). VK5 and VS7 from curly endive and romaine lettuce in the same supermarket, respectively, shared the same PFGE pattern. Of note, VK49 and VX9 from curly endive and tomato samples in City Yantai and Laiyang, respectively, had the same PFGE pattern (Figure 1, Table 1). The two hvKP isolates VH11 and VH1-2 also shared identical PFGE pattern and belonged to ST23, although they were from different cities.

### Antimicrobial susceptibility patterns

As shown in Table 1, the 12 CRE isolates were resistant to all β-lactam antibiotics tested. All *E. coli* and *C. freundii* isolates were resistant to fosfomycin and the veterinary drug, florfenicol. Three isolates, VH1, VK70, and VX9 were resistant to ciprofloxacin, and 2 isolates, VK70 and VK44 showed resistance to amikacin. Notably, all 12 isolates showed multidrug resistance. No isolate was resistant to tigecycline and colistin in this study.

For *E. coli* isolates VS1 and VH3-1 with the same PFGE pattern, different resistance phenotypes were found (Table 1). Although identical PFGE pattern was exist in some of the *C. freundii* isolates, the 5 isolates from different markets or vegetable types showed different resistance phenotypes. The 2 ST23 hvKP isolates with identical PFGE pattern had the same resistance phenotypes, although they were from different cities.

### Other resistance genes in CRE isolates

All *E*. *coli* and *C. freundii* isolates harbored *floR* and *fosA3* genes, responsible for the resistance to florfenicol and fosfomycin, respectively. Both VK70 and VK44 resistant to amikacin, harbored *rmtB*, and no other 16S rRNA methylase gene was found in this study. *qnrB* found in 8 isolates was the most prevalent PMQR gene and all the *C. freundii* and *K. pneumoniae* isolates harbored *qnrB* (Table 1). *oqxAB* was found in 4 CRE isolates, including the 2 *K. pneumoniae* isolates. No *mcr* genes were found in the 12 CRE isolates.

Notably, the 2 *E. coli* isolates VS1 and VS2 with identical PFGE pattern in the same romaine lettuce sample had different genotypes and additional *oqxAB* was present in VS2 (Table 1, Figure 1). Isolates VH3-1 and VS1 sharing the same PFGE pattern and ST type, had the same genotypes. As shown in Table 1, VK5 and VS7 sharing the same PFGE pattern also had the same genotypes. Of note, *C. freundii* isolates VK49 and VX9 from curly endive and tomato samples in City Yantai and Laiyang, respectively, showed different genotypes, although the same PFGE patterns were found between the two isolates (Figure 1, Table 1). The same phenomenon was also observed in the 2 hvKP isolates VH11 and VH1-2.

### Transfer of carbapenemase-encoding resistance genes and plasmid analysis

**Figure 2:**
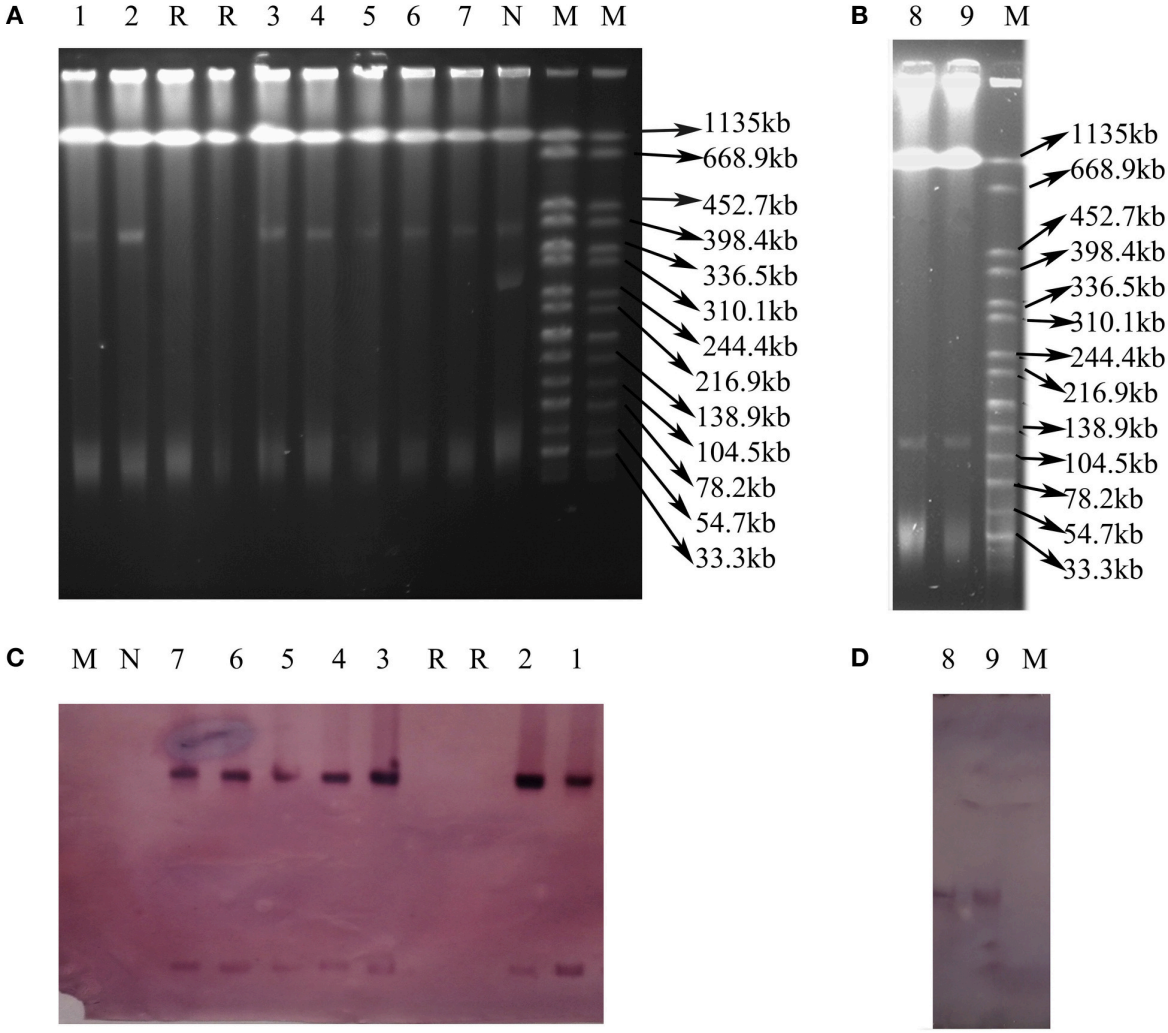
Plasmid analysis of transconjugants carrying *bla*_NDM_ or *bla*_KPC−2_. **(A)** S1 nuclease-PFGE of transconjugants carrying *bla*_NDM_. **(B)** S1 nuclease-PFGE of transconjugants carrying *bla*_KPC−2_. **(C)** Southern blot hybridization with the *bla*_NDM_ probe. **(D)** Southern blot hybridization with the *bla*_KPC−2_ probe. Lane 1–9: VS2T, VS1T, VX9T, VK70T, VH1T, VK49T, VH3-1T, VH1-2T, VH11T. Lane M: H9812; Lane R: Receipt *E. coli* C600; Lane N: *E. coli* isolate without *bla*_NDM_.

Nine transconjugants were successfully obtained from the 12 CRE isolates (VK5, VS7, and VK44 failed) by conjugation experiments. Seven transconjugants carried *bla*_NDM_ and 2 harbored *bla*_KPC−2_, resulting in that all transconjugants were resistant to meropenem, ertapenem, imipenem, cefotaxime, ceftiofur ceftazidime, and ampicillin (Table 2). The results of *S*1 nuclease-PFGE and Southern blot hybridization revealed that all the 7 transconjugants harboring *bla*_NDM_ carried one plasmid (~40 kb) hybridized with *bla*_NDM_ (Figure 2). Interestingly, there were additional bigger bands in all 7 transconjugants in the S1-PFGE and these bands could be also hybridized with the *bla*_NDM_ probe. These bigger bands were the portion of the *bla*_NDM_-carrying plasmids not exposed to S1 nuclease in the S1-PFGE experiment. In both the *bla*_KPC−2_-positive transconjugants, only one plasmid of ~130 kb in size was detected and *bla*_KPC−2_ was confirmed to be on this plasmid as shown in Figure 2. IncX3 replicon type was detected in the 7 plasmids with *bla*_NDM_, while both the *bla*_KPC−2_-positive plasmids belonged to F35:A-:B1 replicon type (Table 2). In the 7 *bla*_NDM_-positive plasmids, co-transfer of resistance to kanamycin and fosfomycin was observed in 1 plasmid, respectively, and *fosA3* was also found in VX9T from *C. freundii* in tomato (Table 2). Co-transfer of resistance to kanamycin was found in both the *bla*_KPC−2_-positive plasmids.

**Table 2:** Characteristics of the nine transconjugants harboring *bla*_NDM_ or *bla*_KPC−2_.

**Donor species and strain**	**Origin of donor**	**Genes**	**MICs (mg/L)**			**Other resistance profiles**	**Plasmid Replicon type (size kb)**	**Plasmid RFLP profiles^a^**	**Donor PFGE patterns^b^**
			**MEM**	**CTX**	**NAL**				
***E. coli***									
VS2T	Romaine lettuce	*bla*_NDM−5_	32	>128	4	AMP, CTF, CAZ, ETP, IPM, STR	X3 (~40)	A1	A
VS1T	Romaine lettuce	*bla*_NDM−5_	>32	64	4	AMP, CTF, CAZ, ETP, IPM, STR	X3 (~40)	A3	A
VK70T	Curly endive	*bla*_NDM−5_	>32	>128	4	AMP, CTF, CAZ, ETP, IPM, STR	X3 (~40)	A4	C
VH1T	Cucumber	*bla*_NDM−5_	>32	>128	4	AMP, CTF, CAZ, ETP, IPM, STR	X3 (~40)	A1	B
VH3-1T	Cucumber	*bla*_NDM−5_	32	64	4	AMP, CTF, CAZ, ETP, IPM, STR	X3 (~40)	A1	A
***C. freundii***									
VX9T	Tomato	*bla*_NDM−1_, *fosA3*	16	128	4	AMP, CTF, CAZ, ETP, IPM, STR FOS	X3 (~40)	A2	D
VK49T	Curly endive	*bla*_NDM−1_	16	64	4	AMP, CTF, CAZ, ETP, IPM, STR, KAN	X3 (~40)	A2	E
***K. pneumoniae***									
VH1-2T	Cucumber	*bla*_KPC−2_	16	32	4	AMP, CTF, CAZ, ETP, IPM, STR, KAN	F35:A-:B1 (~130)	B	F
VH11T	Cucumber	*bla*_KPC−2_	16	16	4	AMP, CTF, CAZ, ETP, IPM, STR, KAN	F35:A-:B1 (~130)	B	F
C600			0.031	0.06	4	STR			

a *RFLP profiles differing by only a few bands (n = 1 ~ 3) were assigned to the same profile*.

b *PFGE patterns of the same donor species differing by only a few bands (n = 1 ~5) were assigned to the same group*.

*AMP, ampicillin; MEM, meropenem; CTX, cefotaxime; CTF, ceftiofur; CAZ, Ceftazidime; ETP, ertapenem; IPM, imipenem; NAL, nalidixic acid; STR, streptomycin; KAN, kanamycin; FOS, fosfomycin*.

**Figure 3:**
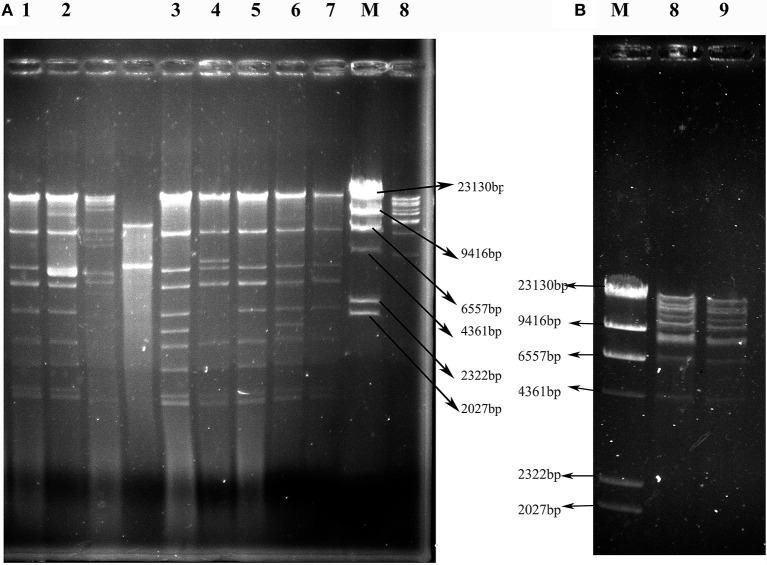
*EcoR*I restriction digestion profiles of plasmids harboring *bla*_NDM_ or *bla*_KPC−2_ genes from transconjugants containing only one plasmid. **(A)** Profiles of IncX3 plasmids. **(B)** Profiles of F35:A-:B1 plasmids. Lanes 1–9: VS2T, VS1T, VX9T, VK70T, VH1T, VK49T, VH3-1T, VH1-2T, and VH11T; Lane M: λ-*Hind*III marker.

As shown in Figure 3, the 7 IncX3 plasmids shared highly similar RFLP profiles. The IncX3 plasmids in *C. freundii* VX9 and VK49 had the same *EcoR*I digestion patterns, although the two isolates were from tomato and curly endive in different cities, respectively (Figure 3, Table 2). The two F35:A-:B1 type *bla*_KPC−2_-positive plasmids from cucumbers in two different cities also shared the same RFLP profiles (Figure 3, Table 2). Notably, the plasmids of 3 *E. coli* isolates (VS2, VH1, and VH3-1) from different vegetable types or cities had identical RFLP profiles (Figure 3, Table 2), and isolates VS2 and VH1 from different vegetables in the same farmer's market had different PFGE patterns.

**Figure 4:**
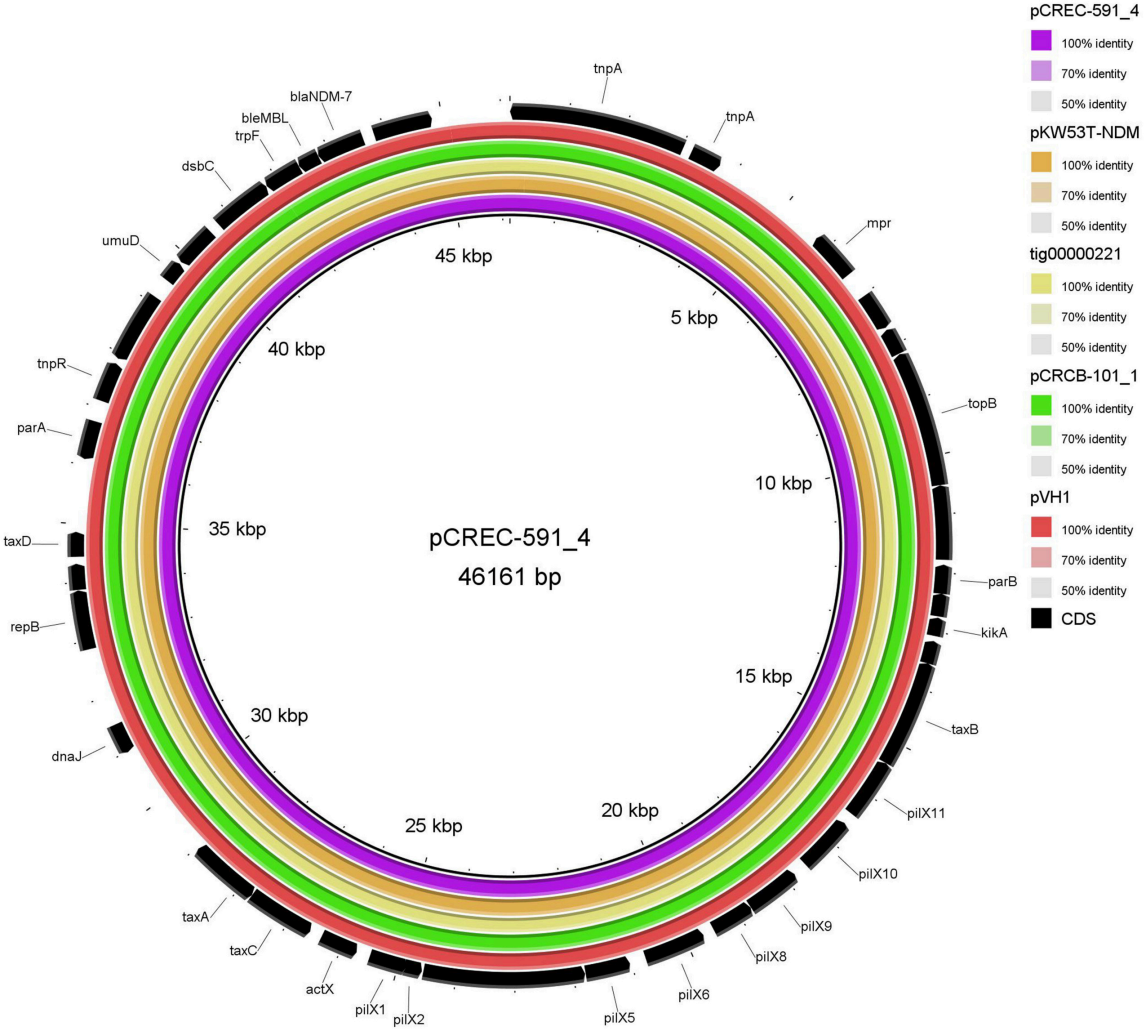
BLASTn-based comparison using BRIG to map complete sequences of plasmids pVH1, pCRCB-101_1, pKW53T-NDM, and tig00000221 against plasmid pCREC-591_4. The innermost ring represents the reference plasmid pCREC-591_4 (GenBank accession number CP024825) from *E. coli* of clinical Peritoneal fluid in South Korea. The orange, yellow, green, and dark red ring represents pKW53T-NDM (KX214669) from *E. coli* of clinical urine in Kuwait, tig00000221 (CP021534) from *E. coli* in the USA, pCRCB-101_1 (CP024820) from *Citrobacter freundii* of clinical Open pus in South Korea and pVH1 (CP028705) from *E. coli* of cucumber in China (this study), respectively. The outermost ring shows annotations of the plasmid pKW53T-NDM.

The IncX3 plasmid pVH1 in VH1T was selected for sequencing in this study, and a single contig that was manually closed to a circle was obtained. pVH1 (accession number CP028705) was 46, 161 bp in length with an average G+C content of 46.7%, and encoded 39 open reading frames (ORF). Beyond *bla*_NDM−5_, the plasmid did not carry any other antibiotic resistance gene and this could account for the phenotypes of transconjugants VH1T. The full-plasmid comparison revealed that pVH1 was closely related to the other IncX3 plasmids in GenBank (Figure 4). Notably, pVH1 from cucumber in Shandong province in this study was identical to plasmids pCREC-591_4 (GenBank accession number CP024825) from *E. coli* of clinical Peritoneal fluid in South Korea and pCRCB-101_1 (CP024820) from *C. freundii* of clinical Open pus in South Korea (Figure 4). Additionally, the three IncX3 plasmids mentioned above were highly similar (99%) to the two *bla*_NDM−7_-bearing plasmids pKW53T-NDM (KX214669) and tig00000221 (CP021534) from clinical *E. coli* isolates in Kuwait and the USA, respectively.

## Discussion

The presence of antibiotic-resistant bacteria in food is a threat to public health, and particular focus has been given to CRE in the food chain. Although *K. variicola* producing OXA-181 and *K. pneumoniae* producing OXA-48 was respectively found in fresh vegetables (Zurfluh et al., 2015b; Touati et al., 2017), both the numbers and locations of the vegetable samples in the two previous reports were relatively few. Recently, only one *E. coli* co-producing NDM-1 and KPC-2 carbapenemases from lettuce was reported in Guangzhou, China. In view of this, we performed a large study to seek CRE isolates from ready-to-eat vegetables purchased at supermarkets and farmer's markets. In the current study, 411 vegetable samples from 18 cities in China were included, and 10 samples, including curly endive, romaine lettuce, cucumber, and tomato were found to harbor NDM and/or KPC-2-producing *Enterobacteriaceae*, implying that these types of vegetables in China may be a source of carbapenemases genes for human microflora and be a threat to human, as previous studies identifying vegetables as a possible route for the dissemination of resistance genes in the community (Zurfluh et al., 2015a,b; Randall et al., 2017). This also confirmed previous finding that *bla*_KPC−2_ and *bla*_NDM_ were the key genes mediating the development of carbapenem resistance phenotypes in CRE in clinical settings in China (Zhang et al., 2017). The highest detection rate of CRE was found in curly endive (14.3%) in this study, different from the three previous reports about CRE in vegetables, indicating that ready-to-eat curly endive should attract more attention because this type of vegetable is often consumed as salads (Francis and O'beirne, 2006). Besides the carbapenemase-producing *K. pneumoniae* and *E. coli* isolates in vegetables reported previously (Touati et al., 2017; Wang et al., 2018), we found *K. pneumoniae* with *bla*_KPC−2_, *E. coli* with *bla*_NDM_, and *C. freundii* with *bla*_NDM_, simultaneously in this study, suggesting various genera of bacteria should be monitored in the future.

Notably, the 2 NDM-producing *E. coli* isolates (VS1 and VS2) found in the same romaine lettuce sample from a farmer's market shared the same PFGE profiles but different genotypes, implying that *E. coli* isolates in vegetables have complex evolutionary process. *E. coli* VH1 with *bla*_NDM−5_ and *K. pneumoniae* VH1-2 with *bla*_KPC−2_ occurred in a single cucumber sample in the same farmer's market as VS1, indicating the environment in farmer's market might further facilitate the spread of CRE isolates. These results also prove that the conventional isolation method that one isolate was selected from each sample will underestimate the detection rate of resistant bacteria, and similar finding was also found in Chinese poultry production (Wang et al., 2017). Of note, *E. coli* VH3-1 and VS1 sharing the same PFGE pattern and ST type were from different vegetable origins and different cities (Table 1, Figure 1), indicating clonal spread exists between different vegetable types or cities in China. The result was confirmed by the 2 *C. freundii* isolates (VK49 and VX9) and the 2 *bla*_KPC−2_-positive *K. pneumoniae* isolates in this study (Figure 1). The reason for this phenomenon was unknown. The contamination of bacteria may occur through irrigation water or animal manure fertilization. Moreover, presence of carbapenem-resistant bacteria could be related to human contamination during manipulation and conservation of vegetables. One *E. coli* isolate VK70 harboring *bla*_NDM−5_ from a curly endive sample belonged to ST167, which has become an internationally disseminated pathogen among human clinical ESBL-producing *E. coli* (Sánchez-Benito et al., 2017) and NDM-producing isolates including NDM-1 (Zhang et al., 2017), NDM-5 (Huang et al., 2016), and NDM-7 (Cuzon et al., 2013). The NDM-5-producing ST167 isolate in vegetable in this study further proved the tight association of ST167 and *bla*_NDM−5_ in China, and is of particular concern. Notably, both the two *bla*_KPC−2_-positive *K. pneumoniae* isolates from cucumbers in this study belonged to ST23 carbapenem-resistant K1 hvKP, which was first reported in patients in China in 2015 (Zhang et al., 2015), and hvKP was more likely to cause liver abscess, sepsis, and invasive infections than classic *K. pneumoniae* strain (Liu et al., 2014; Zhang et al., 2016). Thus, the emergence of ST23 carbapenem-resistant K1 hvKP in fresh vegetables will give rise to further concern for consumer health.

*fosA3*, conferring resistance to fosfomycin, a therapeutic agent effective against common uropathogens in many countries (Falagas et al., 2010), was found in 10 CRE isolates in vegetables, proving the close relationship between *fosA3* and *bla*_NDM_ (Liu et al., 2017). *rmtB* was found in the two CRE isolates resistant to amikacin, a clinically important aminoglycosides drug, proving that 16S rRNA methylase confers high-level resistance to aminoglycosides especially amikacin (Doi and Arakawa, 2007), and *rmtB* is the most prevalent 16S rRNA methylase gene in vegetables, similar to that in clinical isolates in China (Yu et al., 2010). Nine plasmids of the 12 CRE isolates in this study were transferable, representing a threat to human health. The current study showed that the dissemination of *bla*_NDM_ among CRE in ready-to-eat vegetables was mainly mediated by IncX3 conjugative plasmids, consistent with that among clinical CRE strains in China (Yang et al., 2015; Zhang et al., 2017). The bigger bands in the 7 transconjugants (Figure 2) were the portion of the *bla*_NDM_-carrying plasmids not exposed to S1 nuclease in the S1-PFGE experiment according to the findings of a previous study (Barton et al., 1995). So all the 7 transconjugants with *bla*_NDM_ carried only one IncX3 plasmid. The 7 IncX3 plasmids from different genera of bacteria or types of vegetables in this study shared highly similar *EcoR*I digestion patterns and some even had identical patterns, suggesting that horizontal transfer of such mobile elements is also the major mechanism responsible for emergence and transmission of *bla*_NDM_ in vegetables in China, even among various genera of bacteria. The full-plasmid comparison showed that pVH1 with *bla*_NDM−5_ was identical or highly similar (99%) to the other IncX3 plasmids from clinical patients in various countries (Figure 4), suggesting pVH1-like IncX3 plasmids have disseminated around the world and can also spread between clinical isolates and isolates from food. Compared with plasmids harboring *bla*_NDM_, the plasmids carrying *bla*_KPC−2_ were more divergent in clinical settings in China (Zhang et al., 2017), however the two F35:A-:B1 *bla*_KPC−2_-bearing plasmids in *K. pneumoniae* from different cities in our study shared identical backbone structure. This suggests that this type of plasmid might have disseminated among *K. pneumoniae* in vegetables in China and further surveillance of this plasmid should be performed.

In summary, this study reported a high occurrence of CRE in ready-to-eat vegetables in China. *bla*_NDM_ and *bla*_KPC−2_ were the major carbapenemase genes, with *bla*_NDM_ being mediated by highly similar IncX3 plasmids in *E. coli* and *C. freundii*, and *bla*_KPC−2_ being mediated by similar F35:A-:B1 plasmids in *K. pneumoniae*. The sequenced prevalent IncX3 plasmid from cucumber was identical or highly similar (99%) to the other IncX3 plasmids from clinical patients reported in other countries. *E. coli* with *bla*_NDM_ and ST23 type K1 hvKP carrying *bla*_KPC−2_ were observed in a single vegetable sample. Clonal spread also existed between different vegetable types and cities in China. The presence of CRE-producing organisms in the ready-to-eat vegetables is alarming and constitutes a food safety issue. Measures need to be taken to ensure the ready-to-eat vegetables consumer health and further studies are required for monitoring the prevalence of CRE in vegetables in China and other countries. To our knowledge, this is the first report of the high occurrence of CRE in vegetable samples. This is also the first report of either the *C. freundii* carrying *bla*_NDM_, or hvKP harboring *bla*_KPC−2_ in vegetables.

## Author contributions

B-TL and F-JS conceived and designed the experiments. F-JS, X-YZ, and S-WW performed the experiments. F-JS, B-TL, J-JH, and R-DJ analyzed the data. B-TL and F-JS wrote the manuscript.

### Conflict of interest statement

The authors declare that the research was conducted in the absence of any commercial or financial relationships that could be construed as a potential conflict of interest.

## References

[B1] AbuOun M. Stubberfield E. J. Duggett N. A. Kirchner M. Dormer L. Nunez-Garcia J.. (2017). *mcr-1* and *mcr-2* variant genes identified in Moraxella species isolated from pigs in Great Britain from 2014 to 2015. J. Antimicrob. Chemother. 72, 2745–2749. 10.1093/jac/dkx286 29091227 PMC5890717

[B2] Alikhan N. F. Petty N. K. Ben Zakour N. L. Beatson S. A. (2011). BLAST Ring Image Generator (BRIG): simple prokaryote genome comparisons. BMC Genomics 12:402. 10.1186/1471-2164-12-402 21824423 PMC3163573

[B3] Barton B. M. Harding G. P. Zuccarelli A. J. (1995). A general method for detecting and sizing large plasmids. Anal. Biochem. 226, 235–240. 10.1006/abio.1995.1220 7793624

[B4] Belmar Campos C. Fenner I. Wiese N. Lensing C. Christner M. Rohde H.. (2014). Prevalence and genotypes of extended spectrum beta-lactamases in Enterobacteriaceae isolated from human stool and chicken meat in Hamburg, Germany. Int. J. Med. Microbiol. 304, 678–684. 10.1016/j.ijmm.2014.04.012 24856867

[B5] Berger C. N. Sodha S. V. Shaw R. K. Griffin P. M. Pink D. Hand P.. (2010). Fresh fruit and vegetables as vehicles for the transmission of human pathogens. Environ. Microbiol. 12, 2385–2397. 10.1111/j.1462-2920.2010.02297.x 20636374

[B6] Briñas L. Moreno M. A. Zarazaga M. Porrero C. Saenz Y. Garcia M.. (2003). Detection of CMY-2, CTX-M-14, and SHV-12 beta-lactamases in *Escherichia coli* fecal-sample isolates from healthy chickens. Antimicrob. Agents Chemother. 47, 2056–2058. 10.1128/AAC.47.6.2056-2058.2003 12760899 PMC155838

[B7] Buchholz U. Bernard H. Werber D. Bohmer M. M. Remschmidt C. Wilking H.. (2011). German outbreak of *Escherichia coli* O104:H4 associated with sprouts. N. Engl. J. Med. 365, 1763–1770. 10.1056/NEJMoa1106482 22029753

[B8] Carattoli A. Bertini A. Villa L. Falbo V. Hopkins K. L. Threlfall E. J. (2005). Identification of plasmids by PCR-based replicon typing. J. Microbiol. Methods 63, 219–228. 10.1016/j.mimet.2005.03.018 15935499

[B9] Cavaco L. M. Hasman H. Xia S. Aarestrup F. M. (2009). qnrD, a novel gene conferring transferable quinolone resistance in *Salmonella enterica* serovar Kentucky and Bovismorbificans strains of human origin. Antimicrob. Agents Chemother. 53, 603–608. 10.1128/AAC.00997-08 19029321 PMC2630628

[B10] Chen L. Chavda K. D. Al Laham N. Melano R. G. Jacobs M. R. Bonomo R. A.. (2013). Complete nucleotide sequence of a *bla*KPC-harboring IncI2 plasmid and its dissemination in New Jersey and New York hospitals. Antimicrob. Agents Chemother. 57, 5019–5025. 10.1128/AAC.01397-13 23896467 PMC3811408

[B11] Chen L. Chen Z. L. Liu J. H. Zeng Z. L. Ma J. Y. Jiang H. X. (2007). Emergence of RmtB methylase-producing *Escherichia coli* and *Enterobacter cloacae* isolates from pigs in China. J. Antimicrob. Chemother. 59, 880–885. 10.1093/jac/dkm065 17353219

[B12] CLSI (2015a). Performance Standards for Antimicrobial Disk and Dilution Susceptibility Tests for Bacterial Isolated from Animals; Approved Standard-Fourth Edition and Supplement, VET01A4E and VET01S3E. Wayne, PA: Clinical and Laboratory Standards Institute.

[B13] CLSI (2015b). Performance Standards for Antimicrobial Susceptibility Testing; Twenty-Fifth Informational Supplement. CLSI document M100-S25. Wayne, PA: Clinical and Laboratory Standards Institute.

[B14] Cuzon G. Bonnin R. A. Nordmann P. (2013). First identification of novel NDM carbapenemase, NDM-7, in *Escherichia coli* in France. PLoS ONE 8:e61322. 10.1371/journal.pone.0061322 23593461 PMC3625146

[B15] Davis M. A. Baker K. N. Orfe L. H. Shah D. H. Besser T. E. Call D. R. (2010). Discovery of a gene conferring multiple-aminoglycoside resistance in *Escherichia coli*. Antimicrob. Agents Chemother. 54, 2666–2669. 10.1128/AAC.01743-09 20368404 PMC2876372

[B16] Diancourt L. Passet V. Verhoef J. Grimont P. A. Brisse S. (2005). Multilocus sequence typing of *Klebsiella pneumoniae* nosocomial isolates. J. Clin. Microbiol. 43, 4178–4182. 10.1128/JCM.43.8.4178-4182.2005 16081970 PMC1233940

[B17] Doi Y. Arakawa Y. (2007). 16S ribosomal RNA methylation: emerging resistance mechanism against aminoglycosides. Clin. Infect. Dis. 45, 88–94. 10.1086/518605 17554708

[B18] Edelstein M. Sundborger C. Hergens M. P. Ivarsson S. Dryselius R. Insulander M.. (2014). Barriers to trace-back in a salad-associated EHEC Outbreak, Sweden, June 2013. PLoS Curr. 6. 10.1371/currents.outbreaks.80bbab3af3232be0372ea0e904dcd1fe 24944844 PMC4055603

[B19] Falagas M. E. Kastoris A. C. Kapaskelis A. M. Karageorgopoulos D. E. (2010). Fosfomycin for the treatment of multidrug-resistant, including extended-spectrum beta-lactamase producing, Enterobacteriaceae infections: a systematic review. Lancet Infect. Dis. 10, 43–50. 10.1016/S1473-3099(09)70325-1 20129148

[B20] Fang C. T. Lai S. Y. Yi W. C. Hsueh P. R. Liu K. L. Chang S. C. (2007). *Klebsiella pneumoniae* genotype K1: an emerging pathogen that causes septic ocular or central nervous system complications from pyogenic liver abscess. Clin. Infect. Dis. 45, 284–293. 10.1086/519262 17599305

[B21] Francis G. A. O'beirne D. (2006). Isolation and pulsed-field gel electrophoresis typing of *Listeria monocytogenes* from modified atmosphere packaged fresh-cut vegetables collected in Ireland. J. Food Prot. 69, 2524–2528. 10.4315/0362-028X-69.10.2524 17066939

[B22] Friesema I. Sigmundsdottir G. Van Der Zwaluw K. Heuvelink A. Schimmer B. De Jager C.. (2008). An international outbreak of Shiga toxin-producing *Escherichia coli* O157 infection due to lettuce, September–October 2007. Euro Surveill. 13:19065. 10.2807/ese.13.50.19065-en 19087865

[B23] Gautom R. K. (1997). Rapid pulsed-field gel electrophoresis protocol for typing of *Escherichia coli* O157:H7 and other gram-negative organisms in 1 day. J. Clin. Microbiol. 35, 2977–2980. 9350772 10.1128/jcm.35.11.2977-2980.1997 PMC230100

[B24] Huang Y. Yu X. Xie M. Wang X. Liao K. Xue W.. (2016). Widespread dissemination of carbapenem-resistant *Escherichia coli* sequence type 167 strains harboring blaNDM-5 in clinical settings in China. Antimicrob. Agents Chemother. 60, 4364–4368. 10.1128/AAC.00859-16 27114282 PMC4914679

[B25] Johnson T. J. Bielak E. M. Fortini D. Hansen L. H. Hasman H. Debroy C.. (2012). Expansion of the IncX plasmid family for improved identification and typing of novel plasmids in drug-resistant Enterobacteriaceae. Plasmid 68, 43–50. 10.1016/j.plasmid.2012.03.001 22470007

[B26] Jung Y. Jang H. Matthews K. R. (2014). Effect of the food production chain from farm practices to vegetable processing on outbreak incidence. Microb. Biotechnol. 7, 517–527. 10.1111/1751-7915.12178 25251466 PMC4265071

[B27] Lamba M. Graham D. W. Ahammad S. Z. (2017). Hospital wastewater releases of carbapenem-resistance pathogens and genes in urban India. Environ. Sci. Technol. 51, 13906–13912. 10.1021/acs.est.7b03380 28949542

[B28] Leverstein-van Hall M. A. Dierikx C. M. Cohen Stuart J. Voets G. M. Van Den Munckhof M. P. Van Essen-Zandbergen A.. (2011). Dutch patients, retail chicken meat and poultry share the same ESBL genes, plasmids and strains. Clin. Microbiol. Infect. 17, 873–880. 10.1111/j.1469-0691.2011.03497.x 21463397

[B29] Liu B. T. Song F. J. Zou M. Zhang Q. D. Shan H. (2017). High incidence of *Escherichia coli* strains coharboring *mcr-1* and *bla*NDM from chickens. Antimicrob. Agents Chemother. 61:e02347-16. 10.1128/AAC.02347-16 28069644 PMC5328528

[B30] Liu B. T. Wang X. M. Liao X. P. Sun J. Zhu H. Q. Chen X. Y.. (2011). Plasmid-mediated quinolone resistance determinants *oqxAB* and *aac(6')-Ib-cr* and extended-spectrum beta-lactamase gene *bla*CTX-M-24 co-located on the same plasmid in one *Escherichia coli* strain from China. J. Antimicrob. Chemother. 66, 1638–1639. 10.1093/jac/dkr172 21546384

[B31] Liu J. H. Wei S. Y. Ma J. Y. Zeng Z. L. Lu D. H. Yang G. X.. (2007). Detection and characterisation of CTX-M and CMY-2 beta-lactamases among *Escherichia coli* isolates from farm animals in Guangdong Province of China. Int. J. Antimicrob. Agents 29, 576–581. 10.1016/j.ijantimicag.2006.12.015 17314033

[B32] Liu Y. M. Li B. B. Zhang Y. Y. Zhang W. Shen H. Li H.. (2014). Clinical and molecular characteristics of emerging hypervirulent *Klebsiella pneumoniae* bloodstream infections in mainland China. Antimicrob. Agents Chemother. 58, 5379–5385. 10.1128/AAC.02523-14 24982067 PMC4135864

[B33] Liu Y. Y. Wang Y. Walsh T. R. Yi L. X. Zhang R. Spencer J.. (2016). Emergence of plasmid-mediated colistin resistance mechanism MCR-1 in animals and human beings in China: a microbiological and molecular biological study. Lancet Infect. Dis. 16, 161–168. 10.1016/S1473-3099(15)00424-7 26603172

[B34] Lodise T. Ye M. J. Zhao Q. (2017). Prevalence of invasive infections due to carbapenem-resistant *Enterobacteriaceae* among adult patients in U.S. Hospitals. Antimicrob. Agents Chemother. 61:e00228-17. 10.1128/AAC.00228-17 28559271 PMC5527580

[B35] Luo J. Yao X. Lv L. Doi Y. Huang X. Huang S.. (2017). Emergence of *mcr-1* in *Raoultella ornithinolytica* and *Escherichia coli* isolates from retail vegetables in China. Antimicrob. Agents Chemother. 61:e01139-17. 10.1128/AAC.01139-17 28739785 PMC5610531

[B36] Magiorakos A. P. Srinivasan A. Carey R. B. Carmeli Y. Falagas M. E. Giske C. G.. (2012). Multidrug-resistant, extensively drug-resistant and pandrug-resistant bacteria: an international expert proposal for interim standard definitions for acquired resistance. Clin. Microbiol. Infect. 18, 268–281. 10.1111/j.1469-0691.2011.03570.x 21793988

[B37] Mesbah Zekar F. Granier S. A. Marault M. Yaici L. Gassilloud B. Manceau C.. (2017). From farms to markets: gram-negative bacteria resistant to third-generation cephalosporins in fruits and vegetables in a region of north africa. Front. Microbiol. 8:1569. 10.3389/fmicb.2017.01569 28883810 PMC5573783

[B38] Mollet C. Drancourt M. Raoult D. (1997). *rpoB* sequence analysis as a novel basis for bacterial identification. Mol. Microbiol. 26, 1005–1011. 10.1046/j.1365-2958.1997.6382009.x 9426137

[B39] Morrison B. J. Rubin J. E. (2015). Carbapenemase producing bacteria in the food supply escaping detection. PLoS ONE 10:e0126717. 10.1371/journal.pone.0126717 25966303 PMC4429064

[B40] Overbeek R. Olson R. Pusch G. D. Olsen G. J. Davis J. J. Disz T.. (2014). The SEED and the Rapid Annotation of microbial genomes using Subsystems Technology (RAST). Nucleic Acids Res. 42, D206–D214. 10.1093/nar/gkt1226 24293654 PMC3965101

[B41] Petternel C. Galler H. Zarfel G. Luxner J. Haas D. Grisold A. J.. (2014). Isolation and characterization of multidrug-resistant bacteria from minced meat in Austria. Food Microbiol. 44, 41–46. 10.1016/j.fm.2014.04.013 25084643

[B42] Poirel L. Walsh T. R. Cuvillier V. Nordmann P. (2011). Multiplex PCR for detection of acquired carbapenemase genes. Diagn. Microbiol. Infect. Dis. 70, 119–123. 10.1016/j.diagmicrobio.2010.12.002 21398074

[B43] Randall L. P. Lodge M. P. Elviss N. C. Lemma F. L. Hopkins K. L. Teale C. J.. (2017). Evaluation of meat, fruit and vegetables from retail stores in five United Kingdom regions as sources of extended-spectrum beta-lactamase (ESBL)-producing and carbapenem-resistant *Escherichia coli*. Int. J. Food Microbiol. 241, 283–290. 10.1016/j.ijfoodmicro.2016.10.036 27821357

[B44] Rebelo A. R. Bortolaia V. Kjeldgaard J. S. Pedersen S. K. Leekitcharoenphon P. Hansen I. M.. (2018). Multiplex PCR for detection of plasmid-mediated colistin resistance determinants, *mcr-1, mcr-2, mcr-3, mcr-4* and *mcr-5* for surveillance purposes. Euro Surveill. 23:17-00672. 10.2807/1560-7917.ES.2018.23.6.17-00672 29439754 PMC5824125

[B45] Sánchez-Benito R. Iglesias M. R. Quijada N. M. Campos M. J. Ugarte-Ruiz M. Hernandez M.. (2017). *Escherichia coli* ST167 carrying plasmid mobilisable *mcr-1* and *bla*CTX-M-15 resistance determinants isolated from a human respiratory infection. Int. J. Antimicrob. Agents. 50, 285–286. 10.1016/j.ijantimicag.2017.05.005 28599866

[B46] Seo S. Matthews K. R. (2014). Exposure of *Escherichia coli* O157:H7 to soil, manure, or water influences its survival on plants and initiation of plant defense response. Food Microbiol. 38, 87–92. 10.1016/j.fm.2013.08.015 24290631

[B47] Singh-Moodley A. Perovic O. (2016). Antimicrobial susceptibility testing in predicting the presence of carbapenemase genes in Enterobacteriaceae in South Africa. BMC Infect. Dis. 16:536. 10.1186/s12879-016-1858-7 27716102 PMC5050574

[B48] Touati A. Mairi A. Baloul Y. Lalaoui R. Bakour S. Thighilt L.. (2017). First detection of *Klebsiella pneumoniae* producing OXA-48 in fresh vegetables from Bejaia city, Algeria. J. Glob. Antimicrob. Resist. 9, 17–18. 10.1016/j.jgar.2017.02.006 28336324

[B49] Veldman K. Kant A. Dierikx C. Van Essen-Zandbergen A. Wit B. Mevius D. (2014). Enterobacteriaceae resistant to third-generation cephalosporins and quinolones in fresh culinary herbs imported from Southeast Asia. Int. J. Food Microbiol. 177, 72–77. 10.1016/j.ijfoodmicro.2014.02.014 24607424

[B50] Versalovic J. Koeuth T. Lupski J. R. (1991). Distribution of repetitive DNA-sequences in Eubacteria and application to fingerprinting of bacterial genomes. Nucleic Acids Res. 19, 6823–6831. 10.1093/nar/19.24.6823 1762913 PMC329316

[B51] Wachino J. Shibayama K. Kurokawa H. Kimura K. Yamane K. Suzuki S.. (2007). Novel plasmid-mediated 16S rRNA m1A1408 methyltransferase, NpmA, found in a clinically isolated *Escherichia coli* strain resistant to structurally diverse aminoglycosides. Antimicrob. Agents Chemother. 51, 4401–4409. 10.1128/AAC.00926-07 17875999 PMC2168023

[B52] Wang J. Yao X. Luo J. Lv L. Zeng Z. Liu J. H. (2018). Emergence of *Escherichia coli* co-producing NDM-1 and KPC-2 carbapenemases from a retail vegetable, China. J. Antimicrob. Chemother. 73, 252–254. 10.1093/jac/dkx335 29029065

[B53] Wang M. Guo Q. Xu X. Wang X. Ye X. Wu S.. (2009). New plasmid-mediated quinolone resistance gene, *qnrC*, found in a clinical isolate of *Proteus mirabilis*. Antimicrob. Agents Chemother. 53, 1892–1897. 10.1128/AAC.01400-08 19258263 PMC2681562

[B54] Wang Y. Zhang R. Li J. Wu Z. Yin W. Schwarz S.. (2017). Comprehensive resistome analysis reveals the prevalence of NDM and MCR-1 in Chinese poultry production. Nat. Microbiol. 2:16260. 10.1038/nmicrobiol.2016.260 28165472

[B55] Weill F. X. Lailler R. Praud K. Kerouanton A. Fabre L. Brisabois A.. (2004). Emergence of extended-spectrum-beta-lactamase (CTX-M-9)-producing multiresistant strains of *Salmonella enterica* serotype Virchow in poultry and humans in France. J. Clin. Microbiol. 42, 5767–5773. 10.1128/JCM.42.12.5767-5773.2004 15583311 PMC535271

[B56] Wirth T. Falush D. Lan R. Colles F. Mensa P. Wieler L. H.. (2006). Sex and virulence in *Escherichia coli*: an evolutionary perspective. Mol. Microbiol. 60, 1136–1151. 10.1111/j.1365-2958.2006.05172.x 16689791 PMC1557465

[B57] Wu H. Wang Y. Wu Y. Qiao J. Li H. Zheng S.. (2015). Emergence of beta-lactamases and extended-spectrum beta-lactamases (ESBLs) producing *Salmonella* in retail raw chicken in China. Foodborne Pathog. Dis. 12, 228–234. 10.1089/fpd.2014.1859 25658910

[B58] Xie M. Lin D. Chen K. Chan E. W. Yao W. Chen S. (2016). Molecular characterization of *Escherichia coli* strains isolated from retail meat that harbor *bla*CTX-M and *fosA3* Genes. Antimicrob. Agents Chemother. 60, 2450–2455. 10.1128/AAC.03101-15 26856843 PMC4808224

[B59] Yang Q. Fang L. Fu Y. Du X. Shen Y. Yu Y. (2015). Dissemination of NDM-1-producing Enterobacteriaceae mediated by the IncX3-type plasmid. PLoS ONE 10:e0129454. 10.1371/journal.pone.0129454 26047502 PMC4457825

[B60] Yang Y.-Q. Li Y.-X. Lei C.-W. Zhang A.-Y. Wang H.-N. (2018). Novel plasmid-mediated colistin resistance gene *mcr-7.1* in *Klebsiella pneumoniae*. J. Antimicrob. Chemother. 10.1093/jac/dky111. [Epub ahead of print]. 29912417

[B61] Yue L. Jiang H. X. Liao X. P. Liu J. H. Li S. J. Chen X. Y.. (2008). Prevalence of plasmid-mediated quinolone resistance qnr genes in poultry and swine clinical isolates of *Escherichia coli*. Vet. Microbiol. 132, 414–420. 10.1016/j.vetmic.2008.05.009 18573620

[B62] Yu F. Y. Yao D. Pan J. Y. Chen C. Qin Z. Q. Parsons C.. (2010). High prevalence of plasmid-mediated 16S rRNA methylase gene rmtB among *Escherichia coli* clinical isolates from a Chinese teaching hospital. BMC Infect. Dis. 10:184. 10.1186/1471-2334-10-184 20573216 PMC2905422

[B63] Zhang R. Lin D. Chan E. W. Gu D. Chen G. X. Chen S. (2015). Emergence of Carbapenem-resistant serotype K1 hypervirulent *Klebsiella pneumoniae* strains in China. Antimicrob. Agents Chemother. 60, 709–711. 10.1128/AAC.02173-15 26574010 PMC4704206

[B64] Zhang R. Liu L. Zhou H. Chan E. W. Li J. Fang Y.. (2017). Nationwide surveillance of clinical carbapenem-resistant Enterobacteriaceae (CRE) strains in China. EBioMedicine 19, 98–106. 10.1016/j.ebiom.2017.04.032 28479289 PMC5440625

[B65] Zhang Y. Zhao C. Wang Q. Wang X. Chen H. Li H.. (2016). High prevalence of hypervirulent *Klebsiella pneumoniae* infection in China: geographic distribution, clinical characteristics, and antimicrobial resistance. Antimicrob. Agents Chemother. 60, 6115–6120. 10.1128/AAC.01127-16 27480857 PMC5038323

[B66] Zong Z. Zhang X. (2013). *bla*NDM-1-carrying *Acinetobacter johnsonii* detected in hospital sewage. J. Antimicrob. Chemother. 68, 1007–1010. 10.1093/jac/dks505 23288403

[B67] Zurfluh K. Nuesch-Inderbinen M. Morach M. Zihler Berner A. Hachler H. Stephan R. (2015a). Extended-spectrum-beta-lactamase-producing Enterobacteriaceae isolated from vegetables imported from the Dominican Republic, India, Thailand, and Vietnam. Appl. Environ. Microbiol. 81, 3115–3120. 10.1128/AEM.00258-15 25724954 PMC4393435

[B68] Zurfluh K. Poirel L. Nordmann P. Klumpp J. Stephan R. (2015b). First detection of *Klebsiella variicola* producing OXA-181 carbapenemase in fresh vegetable imported from Asia to Switzerland. Antimicrob. Resist. Infect. Control 4:38. 10.1186/s13756-015-0080-5 26448862 PMC4596300

